# A systematic review of the efficiency of recruitment to stroke rehabilitation randomised controlled trials

**DOI:** 10.1186/s13063-019-3991-2

**Published:** 2020-01-10

**Authors:** Kris McGill, Catherine M. Sackley, Jon Godwin, Jodie McGarry, Marian C. Brady

**Affiliations:** 1 Nursing Midwifery and Allied Health Professionals Research Unit, Glasgow Caledonian University, Glasgow, UK; 2 King’s College London, London, UK; 3 Glasgow Caledonian University, Glasgow, UK

**Keywords:** Stroke, Recruitment, Rehabilitation, Randomised controlled trials, Systematic review, Reporting standards

## Abstract

**Introduction:**

Randomised controlled trials (RCTs) that fail to meet their recruitment target risk increasing research waste. Acute stroke RCTs experience notable recruitment issues. The efficiency of recruitment to stroke rehabilitation RCTs has not been explored.

**Aims and objectives:**

To explore recruitment efficiency and the trial features associated with efficient recruitment to stroke rehabilitation RCTs.

**Methods:**

A systematic review of stroke rehabilitation RCTs published between 2005 and 2015 identified in a search of the Cochrane Stroke Group (CSG) Trials Register from 35 electronic databases (e.g. Medline, CINAHL; EMBASE), clinical trial registers, and hand-searching. Inclusion criteria are stroke rehabilitation intervention, delivered by a member of the rehabilitation team, and clinically relevant environment. We extracted data on recruitment efficiency and trial features.

**Results:**

We screened 12,939 titles, 1270 abstracts and 788 full texts, before extracting data from 512 included RCTs (*n* = 28,804 stroke survivor participants). This is the largest systematic review of recruitment to date. A third of stroke survivors screened consented to participate (median 34% (IQR 14–61), on average sites recruited 1.5 participants per site per month (IQR 0.71–3.22), and one in twenty (6% (IQR 0–13) dropped out during the RCT. Almost half (48%) of those screened in the community were recruited compared to hospital settings (27%). Similarly, almost half (47%) those screened at least 6 months after stroke participated, compared to 23% of stroke survivors screened within a month of stroke. When one recruiter screened multiple sites, a median of one stroke survivor was recruited every 2 months compared to more than two per month when there was a dedicated recruiter per site. RCT recruitment was significantly faster per site, with fewer dropouts, for trials conducted in Asia (almost three stroke survivors monthly; 2% dropout) compared to European trials (approximately one stroke survivor monthly; 7% dropout).

**Conclusions:**

One third of stroke survivors screened were randomised to rehabilitation RCTs at a rate of between one and two per month, per site. One in twenty did not complete the trial. Our findings will inform recruitment plans of future stroke rehabilitation RCTs. Limited reporting of recruitment details restricted the subgroup analysis performed.

**Trial registration:**

Prospective Register of Systematic Reviews, registration number CRD42016033067.

## Background

An estimated £132 billion of research funding is wasted each year [1] and recruitment issues are thought to be one of the key contributors to research waste [2]. Successfully meeting recruitment targets is vital to ensure statistically significant results are correctly identified (protect against type 1 and type 2 errors) [3–5] and to ensure accurate interpretation of statistical effect sizes (how effective an intervention has been) [6–9]. Randomised controlled trials (RCTs) experience a range of recruitment inefficiencies, including falling to achieve recruitment targets, exceeding the planned timeframe, requiring recruitment extensions, and early termination [3, 5, 10–14]. Between 1994 and 2002 less than one-third of UK trials funded by Health Technology Assessment (HTA) or Medical Research Council (MRC) met their recruitment targets [10]. In an update, just over half the trials published between 2002 and 2008 met recruitment targets, though the majority recruited at least 80% of their original target [15]. Trials that fail to retain their desired sample throughout the duration of the study also contribute to research waste [16, 17], impacting on the validity, reliability, and generalisability of results [7, 18–20].

RCT reports may lack important recruitment details [21–23] which hinders learning from past recruitment experiences amongst similar trial populations or recruitment contexts. Consolidated Standards of Reporting Trials (CONSORT) provides a checklist for reporting standards and a flow diagram to illustrate the movement of participants through an RCT [21, 22]. Despite this, a review of six major journals found that, although 79% of the included trials reported a CONSORT flow diagram, one-third of these were incomplete and 40% did not include the numbers screened for trial eligibility [22].

Recruitment of stroke survivors to clinical trials is challenging [13, 14, 24]. In the UK more than 100,000 people experience a stroke each year [25, 26] and there are currently 1.2 million people living with stroke-related impairments [27–29]. Stroke rehabilitation aims to maximise recovery, enabling stroke survivors to regain their confidence, independence, and quality of life [30]. In order to ensure stroke survivors receive the best treatment available, RCTs assess the effectiveness of rehabilitation interventions [31]. A systematic review and meta-analysis of 114 large scale (> 300 participants) acute (within 1 month after stroke) pharmacological stroke trials published between 1990 and 2004 reported a mean recruitment rate of 0.79 participants per site per month [13]. An update of this study revealed a median recruitment rate of 0.41 participants per site per month for trials published between 2010 and 2014 [24]. Despite notable difficulties recruiting stroke survivors, and limited improvement over the past 27 years [13, 14], little research has focused on recruitment of stroke survivors to rehabilitation RCTs.

A recent James Lind Alliance priority setting partnership looking at priorities for recruitment research highlighted that improving future recruitment predictions is a key priority [32]. The lack of a recruitment evidence base leads trialists to rely on past experience when anticipating or forecasting recruitment to their new RCTs [33]. Trialists tend to base their predictions on studies with positive recruitment experiences rather than trials that experienced recruitment challenges [34]. A recruitment evidence base would reduce trialists’ reliance on past experiences when planning future stroke rehabilitation RCTs [33].

## Aims and objectives

The aim of our study was to examine recruitment to stroke rehabilitation RCTs published from 2005 to 2015. Specifically, we explored the recruitment efficiency of the RCTs, determined whether specific trial features impacted upon recruitment efficiency, and explored the reporting standards of the included RCTs.

## Methods

### Protocol registration and ethics

Our systematic review protocol was registered with the international Prospective Register of Systematic Reviews (CRD42016033067). No ethical approval was required. Data extracted were securely stored on a password-protected computer and was fully anonymised at the point of data extraction. This systematic review was designed in accordance with the Preferred Reporting Items and Systematic Reviews and Meta-Analysis (PRISMA) reporting guidelines where applicable.

### Inclusion criteria

- Published RCTs (described as RCTs in the paper) that compared a rehabilitation intervention with a control condition (usual care, active control, or an attention control) or another treatment
- Interventions delivered by multi-disciplinary stroke team members (physiotherapist, occupational therapist, speech and language therapist, physician, nurse, or psychologist)
- Participants were stroke survivors (including the control group)
- Interventions were delivered in a stroke rehabilitation location (hospital, outpatient clinic, rehabilitation ward, home, community, nursing home, or support group)

### Identification of studies

We manually hand searched the entire Cochrane Stroke Group (CSG) specialised trials register. We systematically searched for all RCTs published between January 2005 and December 2015. We applied no language restrictions. The CSG trials register contains trials identified from 37 major electronic databases (including MEDLINE, EMBASE, CINAHL, EBSCO, AMED, EMBASE classic, PUBMED, PSYCBITE, PSYCHINFO, and CENTRAL). The register also contains more than 25 clinical trial registers and hand searches of approximately 300 stroke-specific conferences, 150 neurology conferences, 40 neurosurgery conferences, 220 rehabilitation conferences, 60 geriatric conferences, and 96 books. The search strategy used by CSG for MEDLINE has been provided as an example (Additional file 1: Supplement A). For complete details of the search strategies see http://www.dcn.ed.ac.uk/csrg/entity/searchmethods.pdf.

### Outcomes

Recruitment efficiency was the primary outcome and measured in terms of the:

1. Randomisation rate (the number of participants randomised as a percentage of the total number of participants screened for eligibility)
2. Recruitment rate (number of participants randomized by the time spent recruiting in months by site)
3. Dropout (the number of participants that failed to complete the trial as a percentage of the number randomised)

Adherence to the CONSORT reporting standards [35] were evaluated in relation to each trial’s report of the number of participants randomised, location and settings, baseline demographics, dropouts, the period of recruitment and follow-up, source of funding, and sample size calculations. A three-tier system was used to classify the reporting (fully reported, partially reported, and not reported).

### Selection of studies, data extraction and management

All abstracts and full texts were independently screened for eligibility by two reviewers (KMcG and JMcG). Using a pro-forma developed and piloted for this review, data extraction was carried out independently, with reference to the full text and in correspondence with the trialists where possible. Discrepancies were discussed and resolved, with discussion with a third reviewer where required. CONSORT items relating to recruitment were extracted and categorised as “reported”, “partially reported” or “not reported”. Where data extraction items were unavailable from the published article, trialists were contacted via email. Where a reply was not received within 3 weeks, the original email was resent (Additional file 1: Supplement B) . All data were transferred to SPSS for analysis. We extracted data on trial features (Table 1) and relevant CONSORT items (Table 2).

**Table 1:** Recruitment and trial characteristics extracted from included stroke rehabilitation RCTs

	Item extracted	Justification
Recruitment characteristics	Number of patients screened for trial participation	Used to generate randomisation rate outcome
	Number of patients randomised into the trial	Used to generate randomisation rate, recruitment rate and dropout outcomes
	Number of patients who drop out	Used to generate dropout outcome
	Number of sites used for recruitment	Used to generate recruitment rate outcome
	Continent of recruitment	Recruitment has been shown to differ between countries [1, 2]
	Recruitment strategy	The recruitment strategies/methods adopted by trials may affect recruitment efficiency [3]
	Profession of the recruiter	The profession of the recruiter may play a role in willingness of patients to take part in trials [2, 4]. Some professions have been described as ‘gatekeeping’ during the recruitment process [5]
	Number of recruiters per site	The number of people responsible for recruitment may reduce recruitment efficiency [6–9]
Trial characteristics	Publication date	There is evidence to suggest recruitment of stroke survivors for clinical trials is becoming less efficient [10, 11]
	Type of intervention	The treatments on offer can be a motivating factor for potential participants [12, 13]
	Targeted impairment	
	Control condition	
	Stroke survivor residence	Recruitment from a community setting may lead to more efficient recruitment to RCTs [11]. Recruitment of acute stroke survivors within a hospital setting has been highlighted as a problematic recruitment area [10, 11]
	Stage of rehabilitation	
	Funding support	There are potential issues of bias when certain funding bodies are used [14]. Trialists may be influenced by institution pressures to secure funding [15]
	Ethics approval	Trialists are concerned by the impact of research governance on the recruitment process [15, 16]

**Table 2:** CONSORT checklist recruitment data items for RCTs

CONSORT diagram	Inclusion and exclusion criteria	Who enrolled participants
Numbers randomised	Dropouts	Source of funding
Location and settings	Data defining the period of recruitment and follow up	Sample size calculations
Baseline demographics		

Included studies were described in the publications as RCTs. No evaluation of the quality of the randomisation process or other standard risk of bias assessments were undertaken since the purpose of this study was not to consider the effectiveness of a specific intervention [11, 14, 15, 24, 36–39]. Instead, our systematic review adopted an inclusive approach to the RCTs identified allowing a comprehensive evaluation of recruitment to stroke rehabilitation RCTs.

### Data analysis plan

Randomisation rate, recruitment rate and dropout were stratified into trial and recruitment characteristics to allow for subgroup analysis using Kruskal–Wallis tests [40]. Where appropriate, Mann–Whitney *U* tests were used for post hoc analysis in order to explore the significant effect highlighted by the Kruskal–Wallis tests. Trial and recruitment characteristics were only used for subgroup analysis if group sizes were considered balanced [7, 40, 41]. Basic power calculations were referred to, to determine appropriate subgroup analysis (comparing two groups each of N subjects leads to the requirement N > [4/“effect”]^2^ for the detection of a difference at *p* = 0.05 and 80% power, where “effect” is in standard deviations [42]). Bonferroni corrections were manually applied to all post hoc analyses (providing corrected alpha values) in order to control for the effect of multiple comparison testing [43]. The accepted alpha value (0.05) was divided by the number of comparison groups. CONSORT reporting items were evaluated using descriptive statistics and displayed within a table and line graph.

### Distribution of data

Kolmogorov–Smirnov (K-S) analysis was used to statistically test the outcome variables against a normally distributed bell curve [44]. The K-S highlighted non-normative distribution for all three dependent variables (< 0.001). We used non-parametric Kruskal–Wallis tests. Recruitment and trial characteristics were analysed in independent groups, typically with three or more variables. Three of the trial features (publication date, ethics approval, and type of intervention) had two grouping levels and therefore non-parametric Mann–Whitney *U* tests were adopted.

## Results

**Fig. 1:**
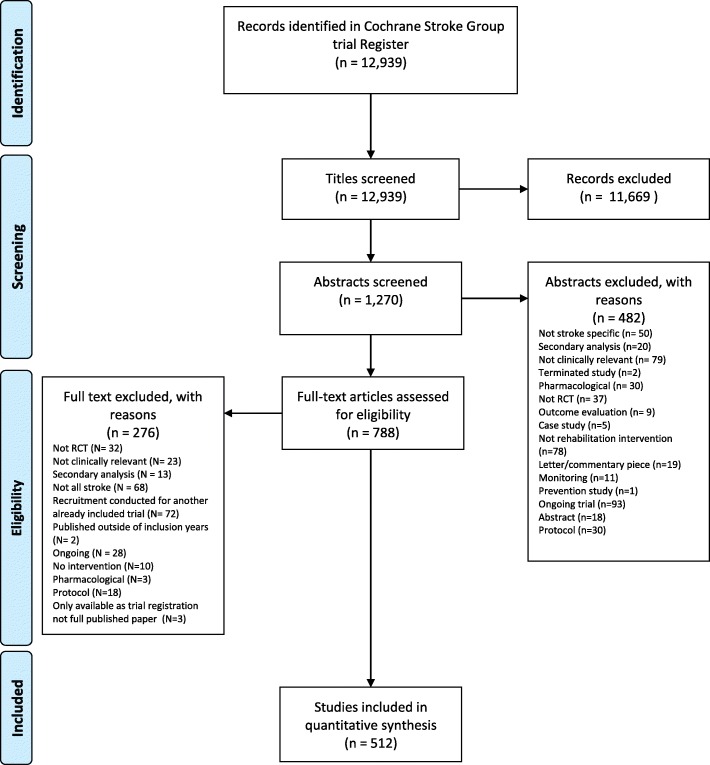
PRISMA diagram showing the flow of systematic identification, screening, inclusion and exclusions of records identified

Of 12,939 titles identified (Fig. 1), 1270 abstracts and 788 full texts were reviewed. We extracted data from 512 stroke rehabilitation RCTs reflecting the randomisation of 28,804 stroke survivors. A range of trial characteristics (Table 1) were used for the all subsequent analyses. A number of trial features were excluded from subgroup analysis because of insufficient data and imbalances across the groups (Table 3), leading to highly uneven group sizes which risked the production of misleading results [40, 41].

**Table 3:** Trial and recruitment characteristics for the included 512 stroke rehabilitation RCTs

	Trial characteristic categories	Number of RCTs in this category (percentage of total, *n* = 512)
Publication date	2009 and before	250 (49%)
	2010 and after	262 (51%)
Trial size	21 or fewer participants	128 (25%)
	22–34 participants	134 (26%)
	35–60 participants	121 (24%)
	61–99 participants	66 (13%)
	100 or more participants	63 (12%)
Stroke survivor residence	Community	100 (19%)
	Hospital	96 (19%)
	Rehabilitation or stroke-specific environment	192 (37%)
	Other^a^	19 (4%)
	Unreported	105 (21%)
Type of intervention	Using a technological aid (any form of equipment to assist the rehabilitation intervention)	270 (59%)
	Not using a technological aid	189 (41%)
	Unreported	53 (10%)
Funding source	Research council	102 (20%)
	Government	65 (13%)
	Charity	51 (10%)
	University	38 (7%)
	No funding	50 (9%)
	Industry^a^	15 (3%)
	Combination^a^	22 (4%)
	Unreported	169 (33%)
Ethics approval	Hospital/health board	191 (37%)
	University	158 (31%)
	Unreported	163 (32%)
Targeted impairment	Arm function	169 (33%)
	Leg function	94 (18%)
	Overall disability	137 (27%)
	Cognitive or vision	61 (12%)
	Unreported	51 (10%)
Stage of rehabilitation	Acute (0–1 month)	82 (16%)
	Acute–subacute	43 (8%)
	Subacute (1–6 months)	62 (12%)
	Subacute–chronic	37 (7%)
	Chronic (> 6 months)	193 (38%)
	Any stage^a^	13 (3%)
	Unreported	82 (16%)
Control condition	Inactive control (form of control that lasts for the same duration as the intervention but does not have a known effect [17])	55 (11%)
	Active control (typically a comparison intervention which is known to have an effect but would not be characterised as standard care [18])	246 (48%)
	Usual care (standard care received)	159 (31%)
	Unreported	52 (10%)
Continent of recruitment	Europe	176 (34%)
	Asia	137 (27%)
	North America	102 (20%)
	Australasia (Australia or New Zealand)^a^	31 (6%)
	Other	66 (13%)
Recruitment strategy	Screening admissions	100 (20%)
	Screening inpatients	81 (16%)
	Screening community dwelling stroke survivors	42 (8%)
	Screening discharge^a^	14 (3%)
	Screening databases^a^	24 (5%)
	Referrals^a^	11 (2%)
	Advertisements^a^	23 (4%)
	Combination of the above^a^	31 (6%)
	Unreported	186 (36%)
Profession of recruiter	Medical professional	47 (9%)
	Allied health professional or nurse	89 (17%)
	Research team member	49 (10%)
	Combination of the above^a^	20 (4%)
	Unreported	307 (60%)
Number of recruiters	One	57 (11%)
	Two or three	59 (11%)
	Four or more	38 (7%)
	Unreported	358 (70%)
Number of recruiters per site	One recruiter covering multiple sites	18 (4%)
	One recruiter per site	47 (9%)
	Between one and two recruiters	38 (7%)
	More than two recruiters	30 (6%)
	Unreported	379 (74%)

^a^Removed from subgroup analysis due to lack of numerical balance increasing risk of producing misleading results

### Recruitment efficiency

The median randomisation rate for stroke rehabilitation trials was 34% (RCTs 321, IQR 47%, range 2% to 100%). The median recruitment rate was 1.5 participants per site per month (RCTs 242, IQR 2.51, range 0.08 to 40). The median dropout rate was 6% (RCTs 414, IQR 13%, range 0% to 83%) Table 4.

Randomisation rate was significantly affected by stroke survivors’ living context, their stage of rehabilitation, the trial’s recruitment strategy, and the number of trial recruiters (Table 5). Post hoc analyses were performed for each of the significant effects. Mann–Whitney *U* tests indicated that recruitment from the community had a significantly higher randomisation rate (Mdn = 0.48) than recruitment from a rehabilitation or stroke-specific environment (Mdn = 0.27; U = 4210.50; *p* = 0.003). Screening community-dwelling stroke survivors had a significantly higher randomisation rate (Mdn = 0.49) than screening admissions (Mdn = 0.22; U = 725; *p* = 0.001). There was a significant difference between recruitment during acute (Mdn = 0.23) and chronic stages after stroke (Mdn = 0.47; U = 2723.5; *p* = 0.001). No other post hoc comparisons were significant. The trial features that did not have a significant effect on recruitment efficiency were RCT publication date, trial size, type of intervention, funding support, ethical approval, target impairment, control condition, recruiter’s profession(s), number of recruiters per site and continent of recruitment (Additional file 1: Supplement C).

**Table 4:** Trial recruitment details and primary recruitment efficiency outcomes (randomisation rate, recruitment rate and dropout) for included stroke rehabilitation RCTs

		RCTs	Median (Mdn)	IQR	Q1	Q3	Min–max
Trial recruitment details	Participants screened	321	126	296	52	348	8–4909
	Participants randomised	512	34	38.75	21.25	60	4–1209
	Recruitment Duration (months)	305	18	19.5	10	29.5	1–152
	Number of recruitment sites	363	1	1	1	2	1–71
Recruitment efficiency	Randomisation rate	321	34%	47%	14%	61%	2–100%
	Recruitment rate	242	1.5	2.51	0.71	3.22	0.08–40
	Dropout	414	6%	13%	0%	13%	0–83%

*RCTs* number of RCTs contributing to analysis, *IQR* interquartile range, *Q1* first quartile, *Q3* third quartile

**Table 5:** Trial and recruitment characteristics that significantly affected randomisation rate for included stroke rehabilitation RCTs

		Trial feature	Kruskal–Wallis	*P*	RCTs		Subgrouping (median)		
Randomisation rate	Trial characteristic	Stroke survivor living context	X^2^(3) = 10.11	*0.018*	239		Community (48%)*		
							General hospital (38%)		
							Stroke-specific environment (27%)		
		Stage of rehabilitation	X^2^(5) = 16.64	*0.002*	292		Acute (23%)		
							Acute–subacute (25%)		
							Subacute (29%)		
							Subacute–chronic (26%)		
							Chronic (48%)*		
	Recruitment characteristic	Recruitment strategy	X^2^(2) = 10.34	*0.006*	167		Screening stroke survivors in the community (49%)*		
							Screening admissions (22%)		
							Screening inpatients (35%)		
		Number of recruiters	X^2^(2) = 6.06	*0.048*	133		Single recruiter (29%)		
							Two or three recruiters (40%)*		
							Four or more recruiters (21%)		

* Best recruitment condition

Kruskal–Wallis = appropriate statistics for Kruskal–Wallis test, *p* = significance level, RCTs = number of RCTs contributing to this finding, X^2^ = chi squared test

Recruitment rate was significantly affected by trial size, targeted impairment, continent of recruitment, and recruiters per site (Table 6). Post hoc analyses were performed to further explore potential effects. Mann–Whitney *U* tests indicated a significantly slower recruitment rate for the trials with 21 or less participants (Mdn = 0.83) when compared to 35–60 (Mdn = 2.45; U = 940.5; *p* < 0.001). There was a significantly faster recruitment rate within RCTs based in Asia (Mdn = 2.68) compared to European RCTs (Mdn = 1.28; U = 1969; *p* > 0.001) and North American RCTs (Mdn = 1.35; U = 706.5; *p* < 0.001). RCTs conducted in Asia recruited at least one more patient per site per month than either European or North American RCTs.

**Table 6:** Trial and recruitment characteristics that significantly affected recruitment rate for included stroke rehabilitation RCTs

		Trial feature	Kruskal–Wallis	*P*	RCTs	Subgrouping (medians)
Recruitment rate	Trial characteristics	Trial size	X^2^(4) = 15.07	*0.005*	242	21 or fewer (0.83)
						22–34 participants (1.53)
						35–60 participants (2.50)*
						61–99 participants (1.7)
						100 ore more (1.62)
		Targeted impairment	X^2^(3) = 14.97	*0.002*	241	Arm function (1.34)
						Leg function (1.84)
						Overall disability 2.16)*
						Cognitive or vision 0.95)
	Recruitment characteristics	Continent of recruitment	X^2^(2) = 24.21	*0.001*	283	Europe (1.28)
						Asia (2.68)*
						North America (1.35)
		Recruiters per site	X^2^(3) = 15.97	*0.001*	122	One recruiter covering multiple sites (0.54)
						One recruiter per site (2.14)*
						Between one and two recruiters per site (1.5)
						More than two recruiters per site (1.9)

* Best recruitment condition

Kruskal-wallis = appropriate statistics for Kruskal–Wallis test, *p* = significance level, RCTs = number of RCTs contributing to this finding

Recruitment rate was significantly slower when recruiters had more than one site (or were not full-time at the site; Mdn = 0.54) when compared to one recruiter per site (Mdn = 2.14; U = 152.5; *p* = 0.001), when compared to between one and two recruiters per site (Mdn = 1.5; U = 174.5; *p* = 0.008), or when compared to more than two recruiters per site (Mdn = 1.94; U = 104.5; *p* < 0.001). There was a significantly faster recruitment rate for interventions targeting gains in overall disability (Mdn = 2.16) compared to trials which targeted improvements in arm function (Mdn = 1.34; U = 2071; *p* = 0.001), cognition or vision (Mdn = 0.95; U = 764.5; *p* = 0.006). There was also a significantly faster recruitment rate for interventions targeting leg function (Mdn = 1.84) when compared to trials that targeted gains in arm function (Mdn = 1.34; U = 1728; *p* = 0.01), cognition or vision (Mdn = 2.16; U = 635; *p* = 0.006). No other significant comparisons were indicated. The trial features that did not have a significant effect were publication date, living context, type of intervention, funding support, ethical approval, stage of stroke rehab, control condition, recruitment strategy, and recruiters per site (Additional file 1: Supplement C).

Dropout rate was significantly affected by publication date, trial size, continent of recruitment, and recruitment strategy (Table 7). Post hoc analysis was then used to further explore effects. Mann–Whitney *U* test indicated a significantly higher dropout rate for RCTs published in or after 2010 (Mdn = 0.09) when compared to RCTs published in 2009 or before (Mdn = 0.03; U = 17,390; *p* = 0.001). There was a significantly lower dropout rate for RCTs with 21 or fewer participants (Mdn = 0) when compared to RCTs with sample sizes of between 22 to 34 participants (Mdn = 0.07; U = 3532; *p* = 0.001), between 35 to 60 participants (Mdn = 0.08; U = 2999; *p* < 0.001), between 60 to 99 participants (Mdn = 0.08; U = 1554; *p* < 0.001), and trials with at least 100 participants (Mdn = 0.008; U = 1350; *p* < 0.001). RCTs conducted in Asia reported significantly lower dropout rates (Mdn = 0.02) when compared to RCTs conducted in Europe (Mdn = 0.07; U = 6694; *p* = 0.002) or North America (Mdn = 0.08; U = 3747; *p* = 0.005). RCTs that screened inpatients experienced significantly lower dropout rates (Mdn = 0.03) than trials that screened hospital admissions (Mdn = 0.09; U = 2788; *p* = 0.015). The trial features that were found to have no significant effect included stroke survivor living context, type of intervention, funding support, ethical approval, targeted impairment, stage of stroke rehabilitation, control condition, profession of recruiter, recruiters per site (Additional file 1: Supplement C). All non-significant post-hoc test results have been provided (Additional file 1: Supplement D).

**Table 7:** Trial and recruitment characteristics that significantly affected dropout for included stroke rehabilitation RCTs

		Trial feature	Kruskal-Wallis	*P*	RCTs	Subgrouping (medians)
Dropout	Trial characteristic	Publication date	X^2^(1) = 10.73	*0.001*	414	2009 or before (3%)*
						2010 or after (9%)
		Trial size	X^2^(4) = 25.38	*< 0.001*	414	21 or fewer (0%)*
						22–34 participants (7%)
						35–60 participants (8%)
						61–99 participants (8%)
						100 ore more (8%)
	Recruitment characteristic	Continent of recruitment	X^2^(2) = 11.91	*0.003*	354	Europe (7%)
						Asia (2%)
						North America (8%)
		Recruitment Strategy	X^2^(2) = 6.09	*0.048*	205	Screening stroke survivors in the community (9%)
						Screening admissions (8%)
						Screening inpatients (3%)*

* Least dropout

Kruskal–Wallis = appropriate statistics for Kruskal–Wallis test, *p* = significance level, RCTs = number of RCTs contributing to this finding

### Consort reporting standards

**Fig. 2:**
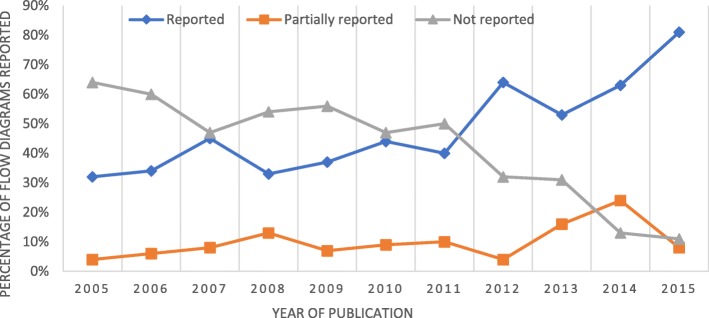
Reporting, partial reporting and not reporting CONSORT diagram in published reports of stroke rehabilitation RCTs published between 2005 and 2015

The CONSORT items related to recruitment (CONSORT diagram, inclusion and exclusion criteria, who enrolled participants, numbers randomised, dropouts, source of funding, location and settings, data defining the period of recruitment and follow up, sample size calculations, baseline demographics) that were extracted from the 512 RCTs included in the systematic review are presented (Table 8 and Fig. 2). The percentage of RCTs fully reporting the CONSORT diagram improved from 32% to 81% between 2005 and 2010. The percentage of trial reports that did not include a CONSORT diagram fell steadily from 64% to 11% over the same period (Fig. 2).

**Table 8:** Number and percentage of RCTs that included each CONSORT reporting item associated with recruitment

Consort reporting item	Data availability	Fully reported	Poorly reported	Not reported
Consort diagram	512	240 (47%)	48 (9%)	224 (44%)
Inclusion and exclusion criteria	459	439 (96%)	4 (1%)	16 (3%)
Who enrolled participants	459	86 (19%)		373 (81%)
Number randomised	459	459 (100%)		
Dropout	459	388 (85%)		71 (15%)
Funding	459	319 (69%)		140 (31%)
Location and setting	459	395 (86%)	57 (13%)	6 (1%)
Period spent with recruitment and follow-up	459	194 (42%)		266 (58%)
Sample size calculations	459	129 (28%)		330 (72%)
Baseline demographics	459	435 (95%)	6 (1%)	18 (4%)

% = percentage of 512 included trials

## Discussion

We explored the recruitment efficiency of stroke rehabilitation RCTs over a 10-year period. We found that one-third of stroke survivors screened were randomised into the trial, between one and two were recruited per site per month, and one in twenty stroke survivors randomised did not complete the trial. Stroke survivors were recruited most efficiently from i) the community, ii) utilising two or three recruiters per site and iii) within the chronic stage of recovery (more than 6 months after stroke). The slowest recruitment rate was experienced by i) the smallest RCTs (less than 21 participants), ii) RCTs conducted in Europe or North America and iii) RCTs using one recruiter to cover multiple sites. The lowest reported dropout was experienced by RCTs i) published before 2009, ii) conducted in Asia and iii) with the smallest sample sizes (less than 21 participants).

Our systematic review illustrates that stroke rehabilitation RCTs have intensive randomisation rates and relatively slow recruitment [3–5, 10, 13, 14]. Interestingly, the recruitment rate we observed for stroke rehabilitation RCTs was faster than the rate experienced by acute stroke RCTs [13, 14]. Stroke rehabilitation RCTs may have more freedom to recruit stroke survivors from across different contexts because of the multiple locations and environments in which rehabilitation takes place. Stroke survivors who are no longer in an acute setting may be more able to attend and commit to clinical trials. However, although it may be more efficient to recruit chronic stroke survivors living at home, stroke rehabilitation improvements are known to be greatest within the early stages following stroke onset. Thus, recruitment plans should be balanced with the trial objectives.

### CONSORT reporting standards

Almost all included RCTs reported inclusion and exclusion criteria, numbers randomised, location and setting of trial, baseline demographics, and dropout. Less than a fifth reported the staff members involved with enrolment and less than half reported the recruitment duration. A priori sample size calculations were reported in less than a third of included RCTs and one-third of RCTs did not report their funders. CONSORT diagrams were fully reported by less than half of stroke rehabilitation RCTs, reflecting an improvement in trial reporting over the past 10 years [22]. This improvement came after the first major CONSORT update in 2010 [35] and is likely to reflect changes in editorial requirements from journals.

### Faster recruitment and less dropout for RCTs conducted in Asia

RCTs conducted in Asia recruited one more patient per site per month and experienced 5% less dropout when compared to RCTs conducted in Europe or America. Cultural differences may have contributed to this effect, with participants in Asian countries being motivated more by societal collectivism when compared to western individualistic societies [45–48]. Collectivist societies tend to place importance on social identity and societal benefit [47]. In comparison, individualistic ideologies place more importance on self-importance and self-gain [48]. Stroke survivors based in Asian countries may be more willing to take part in RCTs because they find value in the potential societal benefits. Another potential influencing factor is the difference in medical staff–patient relationships [49–52]. Southeast Asia tends to have a more authoritarian approach to this interaction, with medical staff directing patients with little feedback [49, 53]. Furthermore, recruitment for RCTs conducted in Asia may be considered unethical by western standards, potentially affecting recruitment rate by reducing the need for some safeguards associated with modern research governance [54–57]. Of 9488 Chinese RCTs published between 2013 and 2016 across 12 nursing journals, informed consent was reported in only 51.8% of RCTs, and written consent in only 27.4%. Potentially, ethical processes for Asian trials could be improved by targeted research governance initiatives, leading to more trials using informed consent.

### Recruitment from the community

For stroke rehabilitation RCTs community-based trials appear to have the most efficient recruitment. During the chronic stage of recovery stroke survivors may be in a better position to be approached regarding trial participation because the immediate psychological and physical implications for the stroke survivor have subsided [25, 26] and the most intensive period of standard rehabilitation is likely to have been completed [25, 26]. By recruiting chronic stroke survivors within the community there may be more time to form relationships and this approach could increase trust during the recruitment conversation [58, 59]. Recruitment forecasting should carefully consider the location of recruitment and the stage of rehabilitation when allocating recruitment time.

### Recruitment staffing levels

Having one recruiter covering multiple sites who has a high work load may affect the ability to identify, screen, and approach potentially eligible participants for recruitment [10]. This is potentially contributed to by under-resourcing during trial planning [33], affecting the amount of time the clinical staff have to recruit [60]. However, the evidence did not support the notion that more recruiters led to more efficient recruitment. This may be caused by the lack of personal responsibility for a site’s recruitment target and accountability for recruitment failures. This may be caused by diffusion of responsibility [61–63] where individuals do not feel personally responsible for a task owing to the belief that someone else will do it, and this is effected by how competent an individual feels in their ability to complete a task [64].

### Strengths of our systematic review

We adopted rigorous methods to search, screen, extract, and analyse all included stroke rehabilitation RCTs. The comprehensive nature of our search strategy supported the inclusion of all stroke rehabilitation RCTs published between 2005 and 2015. Two researchers independently screened all identified trials to ensure that all decisions were independently checked. Our systematic review included 512 stroke rehabilitation RCTs, making it the largest systematic review of trial recruitment conducted to date. Our large sample size ensured that our analysis had the statistical power to confidently protect against type 1 and type 2 errors and supported subgroup analysis of the trial features that could be important for recruitment efficiency. In total, additional information was provided by 177 authors, substantially increasing the availability of data for subgroup analysis.

### Limitations of the systematic review

RCTs were included if the author described randomization methods. Inclusion of RCTS without quality appraisal was intentional in order to include and evaluate stroke rehabilitation RCTs regardless of quality. This allowed for the exploration of recruitment to all stroke rehabilitation RCTs, creating a well-rounded picture of the body of research. The ability to include RCTs not published in English could have contributed to the investigation of more trial features that impact recruitment efficiency. Unfortunately, the lack of reporting of recruitment details by RCTs governed some of the subgroup analysis that could be conducted. Some trial features that could have provided an insight into what makes for successful recruitment may have been missed. We did not extract individual participant level data on stroke severity (NIHSS for example) as our analysis was based only on group summary data. It is possible that stroke profile factors (such as severity of stroke, ability to consent, communication impairment) are important factors for trial participation at an individual participant level but these were not examined in our review.

### Implications

The output from this study provides an evidence base for stroke rehabilitation trialists planning future RCTs. This evidence base could allow for more efficient recruitment to stroke rehabilitation RCTs through more accurate recruitment forecasting and knowledge of what trial features appear to facilitate efficient recruitment. We hope in future to observe a reduction in a) the number of trials that do not reach their target sample size, b) the production of inaccurate and unusable outcomes and c) the number of costly extensions and trial terminations. Better recruitment to stroke rehabilitation trials would lead to more accurate assessments of experimental treatment effects [3–5, 65], contributing to a more robust evidence base for stroke rehabilitation clinicians and better rehabilitation for stroke survivors.

## Conclusions

Stroke rehabilitation RCTs experience recruitment inefficiencies including intensive screening rates and slow recruitment. RCTs recruiting stroke survivors living at home several months after their stroke had the highest level of recruitment efficiency. One recruiter based at a single site was the most efficient approach while a single recruiter covering multiple sites was the least efficient. Trials conducted in Asia experience significantly faster recruitment and fewer dropouts. Our findings will assist recruitment planning of future stroke rehabilitation RCTs contributing to a reduction in research waste amongst future stroke rehabilitation trials.

## Supplementary information

**Additional file 1.** Supplment A: example cochrane group search strategy for MEDLINE B: Note on author contact C: Non-sig effects of trial features D: All non-sig post-hoc results.

## Data Availability

The extracted data used and analysed during the current study are available from the corresponding author upon request.

## Abbreviations

BMJ: British Medical Journal
CONSORT: Consolidation of Standards for Reporting Trials
CSG: Cochrane Stroke Group
HTA: Health Technology Assessment
JAMA: Journal of the American Medical Association
MRC: Medical Research Council
RCT: Randomised controlled trial
SD: Standard deviation
