# Supplementary material for: A systematic review of the efficiency of recruitment to stroke rehabilitation randomised controlled trials
Source: Trials. 2020 Jan 10;21:68. doi: 10.1186/s13063-019-3991-2 (PMC6954526; doi:10.1186/s13063-019-3991-2)
Supplement: Supplementary file 1 — Additional file 1. Supplment A: example cochrane group search strategy for MEDLINE B: Note on author contact C: Non-sig effects of trial features D: All non-sig post-hoc results. [file 13063_2019_3991_MOESM1_ESM.docx]

**Supplementary material**

*Supplement A*

Example Cochrane Stroke Group search strategy for MEDLINE

1. cerebrovascular disorders/
2. exp basal ganglia cerebrovascular disease/
3. exp brain ischemia/
4. exp carotid artery diseases/
5. stroke/
6. exp brain infarction/
7. exp cerebrovascular trauma/
8. hypoxia-ischemia, brain/
9. exp intracranial arterial diseases/
10. exp intracranial arteriovenous malformations/
11. exp "Intracranial Embolism and Thrombosis"/
12. exp intracranial hemorrhages/
13. vasospasm, intracranial/
14. vertebral artery dissection/
15. aneurysm, ruptured/ and exp brain/
16. brain injuries/
17. brain injury, chronic/
18. exp carotid arteries/
19. endarterectomy, carotid/
20. *heart septal defects, atrial/ or foramen ovale, patent/
21. *atrial fibrillation/
22. (stroke or poststroke or post-stroke or cerebrovasc$ or brain vasc$ or cerebral vasc$ or cva$ or apoplex$ or isch?emi$ attack$ or tia$1 or neurologic$ deficit$ or SAH or AVM).tw.
23. ((brain$ or cerebr$ or cerebell$ or cortical or vertebrobasilar or hemispher$ or intracran$ or intracerebral or infratentorial or supratentorial or MCA or anterior circulation or posterior circulation or basal ganglia) adj5 (isch?emi$ or infarct$ or thrombo$ or emboli$ or occlus$ or hypox$ or vasospasm or obstruction or vasculopathy)).tw.
24. ((lacunar or cortical) adj5 infarct$).tw.
25. ((brain$ or cerebr$ or cerebell$ or intracerebral or intracran$ or parenchymal or intraventricular or infratentorial or supratentorial or basal gangli$ or subarachnoid or putaminal or putamen or posterior fossa) adj5 (haemorrhage$ or hemorrhage$ or haematoma$ or hematoma$ or bleed$)).tw.
26. ((brain or cerebral or intracranial or communicating or giant or basilar or vertebral artery or berry or saccular or ruptured) adj5 aneurysm$).tw.
27. (vertebral artery dissection or cerebral art$ disease$).tw.
28. ((brain or intracranial or basal ganglia or lenticulostriate) adj5 (vascular adj5 (disease$ or disorder or accident or injur$ or trauma$ or insult or event))).tw.
29. ((isch?emic or apoplectic) adj5 (event or events or insult or attack$)).tw.
30. ((cerebral vein or cerebral venous or sinus or sagittal) adj5 thrombo$).tw.
31. (CVDST or CVT).tw.
32. ((intracranial or cerebral art$ or basilar art$ or vertebral art$ or vertebrobasilar or vertebral basilar) adj5 (stenosis or isch?emia or insufficiency or arteriosclero$ or atherosclero$ or occlus$)).tw.
33. ((venous or arteriovenous or brain vasc$) adj5 malformation$).tw.
34. ((brain or cerebral) adj5 (angioma$ or hemangioma$ or haemangioma$)).tw.
35. carotid$.tw.
36. (patent foramen ovale or PFO).tw.
37. ((atrial or atrium or auricular) adj fibrillation).tw.
38. asymptomatic cervical bruit.tw.
39. exp aphasia/ or anomia/ or hemiplegia/ or hemianopsia/ or exp paresis/ or deglutition disorders/ or dysarthria/ or pseudobulbar palsy/ or muscle spasticity/
40. (aphasi$ or apraxi$ or dysphasi$ or dysphagi$ or deglutition disorder$ or swallow$ disorder$ or dysarthri$ or hemipleg$ or hemipar$ or paresis or paretic or hemianop$ or hemineglect or spasticity or anomi$ or dysnomi$ or acquired brain injur$ or hemiball$).tw.
41. ((unilateral or visual or hemispatial or attentional or spatial) adj5 neglect).tw.
42. or/1-41
43. Randomized Controlled Trials as Topic/
44. random allocation/
45. Controlled Clinical Trials as Topic/
46. control groups/
47. clinical trials as topic/ or clinical trials, phase i as topic/ or clinical trials, phase ii as topic/ or clinical trials, phase iii as topic/ or clinical trials, phase iv as topic/
48. Clinical Trials Data Monitoring Committees/
49. double-blind method/
50. single-blind method/
51. Placebos/
52. placebo effect/
53. cross-over studies/
54. Multicenter Studies as Topic/
55. Therapies, Investigational/
56. Drug Evaluation/
57. Research Design/
58. Program Evaluation/
59. evaluation studies as topic/
60. randomized controlled trial.pt.
61. controlled clinical trial.pt.
62. (clinical trial or clinical trial phase i or clinical trial phase ii or clinical trial phase iii or clinical trial phase iv).pt.
63. multicenter study.pt.
64. (evaluation studies or comparative study).pt.
65. meta analysis.pt.
66. meta-analysis as topic/
67. random$.tw.
68. (controlled adj5 (trial$ or stud$)).tw.
69. (clinical$ adj5 trial$).tw.
70. ((control or treatment or experiment$ or intervention) adj5 (group$ or subject$ or patient$)).tw.
71. (surgical adj5 (group$ or subject$ or patient$)).tw.
72. (quasi-random$ or quasi random$ or pseudo-random$ or pseudo random$).tw.
73. ((multicenter or multicentre or therapeutic) adj5 (trial$ or stud$)).tw.
74. ((control or experiment$ or conservative) adj5 (treatment or therapy or procedure or manage$)).tw.
75. ((singl$ or doubl$ or tripl$ or trebl$) adj5 (blind$ or mask$)).tw.
76. (coin adj5 (flip or flipped or toss$)).tw.
77. latin square.tw.
78. versus.tw.
79. (cross-over or cross over or crossover).tw.
80. placebo$.tw.
81. sham.tw.
82. (assign$ or alternate or allocat$ or counterbalance$ or multiple baseline).tw.
83. controls.tw.
84. (treatment$ adj6 order).tw.
85. (meta-analy$ or metaanaly$ or meta analy$ or systematic review or systematic overview).tw. 86. or/43-85
87. 42 and 86
88. 87 not exp animals/
89. 87 and humans/
90. 88 or 89

*Supplement B*

Non-significant effects of trial features for randomisation rate, recruitment rate, and dropout rate

|  |  | **Trial feature** | **Kruskal-Wallis** | ***p*** | RCT |
| --- | --- | --- | --- | --- | --- |
| **Randomisation rate** | Trial Characteristic | Publication date | X^2^(1)= .1 | *0.318* | 321 |
|  |  | Trial size | X^2^(4)= 5.5 | *0.240* | 321 |
|  |  | Type of intervention | X^2^(1)= 2.19 | *0.139* | 315 |
|  |  | Funding support | X^2^(6)= 6.42 | *0.378* | 257 |
|  |  | Ethical approval | X^2^(2)= 1 | *0.606* | 248 |
|  |  | Targeted impairment | X^2^(3)= 5.62 | *0.132* | 315 |
|  |  | Control condition | X^2^(2)= .98 | *0.612* | 315 |
|  | Recruitment characteristic | Profession of recruiter | X^2^(3)= 2.02 | *0.568* | 171 |
|  |  | Recruiters per site | X^2^(3)= 1.78 | *0.620* | 116 |
|  |  | Continent of recruitment | X^2^(2)= 5.06 | *0.08* | 283 |
| **Recruitment rate** | Trial characteristic | Publication date | X^2^(1)= .208 | *0.648* | 242 |
|  |  | Living context | X^2^(2)= 1.495 | *0.474* | 221 |
|  |  | Type of intervention | X^2^(1)= .01 | *0.917* | 241 |
|  |  | Funding support | X^2^(6)= 9.97 | *0.126* | 206 |
|  |  | Ethical approval | X^2^(2)= 2.92 | *0.232* | 193 |
|  |  | Stage of stroke rehab | X^2^(5)= 9.98 | *0.076* | 226 |
|  |  | Control condition | X^2^(2)= 2.9 | *0.235* | 241 |
|  | Recruitment characteristic | Recruitment strategy | X^2^(2)= .49 | *0.782* | 157 |
|  |  | Profession of recruiter | X^2^(3)= 2.02 | *0.232* | 151 |
|  |  | Recruiters per site | X^2^(2)= 2.92 | *0.232* | 122 |
| **Dropout** | Trial characteristic | Living context | X^2^(2)= .237 | *0.888* | 344 |
|  |  | Type of intervention | X^2^(1)= .96 | *0.327* | 395 |
|  |  | Funding support | X^2^(6)= 6.14 | *0.407* | 310 |
|  |  | Ethical approval | X^2^(2)= 1.24 | *0.537* | 309 |
|  |  | Targeted impairment | X^2^(3)= 3.94 | *0.268* | 396 |
|  |  | Stage of stroke rehab | X^2^(5)= 9.41 | *0.094* | 373 |
|  |  | Control condition | X^2^(2)= 1.67 | *0.433* | 395 |
|  | Recruitment characteristic | Profession of recruiter | X^2^(3)= 3.62 | *0.306* | 198 |
|  |  | Number of recruiters | X^2^(2)= 1.34 | *0.511* | 149 |
|  |  | Recruiters per site | X^2^(3)= .98 | *0.805* | 128 |

(key: *p* = significant level, Kruskal-wallis = relevant KW test statistics, RCT = number of RCTs included in analysis)

*Supplement B*

Note on author contact

An attempt was made to contact all primary research trialists, however, some contact email addresses were not provided for RCTs, some email addresses were no longer valid (69 email addresses), and new emails could not be located. Emails were successfully sent to 443 authors for additional details regarding their trial. The additional details requested corresponded to items required for primary and subgroup analysis that were unavailable in the published report. Additional data items were reported by 177 authors in response to the request for the information.

*Supplement C*

All non-significant post-hoc test results

|  |  |  | **Mdn** | ***U*** | ***p*** |
| --- | --- | --- | --- | --- | --- |
| **Randomisation rate** | **Stroke survivor living context** | Community vs general hospital | 0.48 vs 0.38 | 2124 | 0.243 |
|  |  | General Hospital vs stroke specific environment | 0.38 vs 0.27 | 3571 | 0.108 |
|  | **Stage of rehabilitation** | Acute vs acute – subacute | 0.23 vs 0.25 | 876.5 | 0.761 |
|  |  | Acute vs subacute | 0.23 vs 0.29 | 1136.5 | 0.535 |
|  |  | Acute vs subacute – chronic | 0.23 vs 0.26 | 648.5 | 0.519 |
|  |  | Acute – subacute vs subacute | 0.25 vs 0.29 | 614 | 0.428 |
|  |  | Acute – subacute vs subacute – chronic | 0.25 vs 0.26 | 353 | 0.45 |
|  |  | Acute – subacute vs chronic | 0.25 vs 0.47 | 1430.5 | 0.003 |
|  |  | Subacute vs subacute – chronic | 0.29 vs 0.26 | 525.5 | 0.879 |
|  |  | Subacute vs chronic | 0.29 vs 0.47 | 2264.5 | 0.03 |
|  |  | Subacute – chronic vs chronic | 0.26 vs 0.47 | 1343 | 0.105 |
|  | **Recruitment strategy** | Screening admissions vs screening inpatients | 0.22 vs 0.35 | 2014 | 0.357 |
|  |  | Screening community vs screening inpatients | 0.49 vs 0.35 | 727 | 0.035 |
|  | **Number of recruiters** | Single recruiter vs two or three recruiters | 0.29 vs 0.40 | 934.5 | 0.076 |
|  |  | Single recruiter vs four or more recruiters | 0.29 vs 0.21 | 677 | 0.448 |
|  |  | Two or three recruiters and four or more recruiters | 0.40 vs 0.21 | 692 | 0.025 |
| **Recruitment rate** | **Trial size** | <21 vs 22 – 34 | 0.83 vs 1.53 | 940.5 | 0.008 |
|  |  | <21 vs 61 – 99 | 0.83 vs 1.7 | 618.5 | 0.019 |
|  |  | <21 vs >100 | 0.83 vs 1.62 | 553 | 0.008 |
|  |  | 22 – 34 vs 35 – 60 | 1.53 vs 2.50 | 1469.5 | 0.117 |
|  |  | 22 – 34 vs 61 – 99 | 1.53 vs 1.7 | 1193.5 | 0.514 |
|  |  | 22 – 34 vs >100 | 1.53 vs 1.62 | 1179.5 | 0.736 |
|  |  | 35 – 60 vs 61 – 99 | 2.50 vs 1.7 | 1044 | 0.448 |
|  |  | 60 – 99 and >100 | 2.50 vs 1.62 | 952.5 | 0.291 |
|  | **Targeted impairment** | Arm function vs cognitive or vision | 1.34 vs 0.95 | 1479.5 | 0.325 |
|  |  | Leg function vs overall disability | 1.84 vs 2.16 | 1567 | 0.932 |
|  | **Continent of recruitment** | Europe vs North America | 1.28 vs 1.35 | 2395.5 | 0.355 |
|  | **Number of recruiters per site** | One per site vs two | 2.14 vs 1.5 | 541 | 0.126 |
|  |  | One per site vs two | 2.14 vs 1.9 | 581 | 0.981 |
|  |  | One or two per site vs two or more | 1.5 vs 1.9 | 416 | 0.151 |
| **Dropout rate** | **Trial size** | 22 – 34 participants vs 35 – 60 | 0.07 vs 0.08 | 5466 | 0.361 |
|  |  | 22 – 34 vs 61 – 99 | 0.07 vs 0.08 | 2978.5 | 0.282 |
|  |  | 22 – 34 vs >100 | 0.07 vs 0.08 | 2648.5 | 0.345 |
|  |  | 35 – 60 vs 61 – 99 | 0.08 vs 0.08 | 3013.5 | 0.771 |
|  |  | 35 – 60 vs >100 | 0.08 vs 0.08 | 2695 | 0.895 |
|  |  | 61 – 99 vs >100 | 0.08 vs 0.08 | 1519.5 | 0.931 |
|  | **Continent of recruitment** | Europe vs North America | 0.07 vs 0.08 | 6575.5 | 0.580 |
|  | **Recruitment strategy** | Screening admissions vs screening community | 0.08 vs 0.09 | 1514.5 | 0.505 |
|  |  | Screening inpatients vs screening community | 0.03 vs 0.09 | 1200 | 0.2 |

(Key: Mdn = median, U = Mann-Whitney U value, *p* = significance level)
